# The population structure of *Glossina fuscipes fuscipes* in the Lake Victoria basin in Uganda: implications for vector control

**DOI:** 10.1186/1756-3305-5-222

**Published:** 2012-10-04

**Authors:** Chaz Hyseni, Agapitus B Kato, Loyce M Okedi, Charles Masembe, Johnson O Ouma, Serap Aksoy, Adalgisa Caccone

**Affiliations:** 1 Department of Ecology and Evolutionary Biology, Yale University, 21 Sachem Street, New Haven, CT, USA; 2 Department of Biology, Makerere University, College of Natural Sciences, School of Bio-Sciences, P.O. Box 7062, Kampala, Uganda; 3 Creation of Sustainable Tsetse and Trypanosomiasis Free Areas in Uganda project, Ministry of Agriculture, Animal Industry and Fisheries, P.O. Box 102, Entebbe, Uganda; 4 National Livestock Resources Research Institute, Tororo, Uganda, P.O. Box 96, Tororo, Uganda; 5 Trypanosomiasis Research Centre, Kenya Agricultural Research Institute, P.O. Box 362–00902, Kikuyu, Kenya; 6 Yale School of Public Health, Yale University, 60 College Street, New Haven, CT, USA

**Keywords:** *Glossina fuscipes fuscipes*, Tsetse, Tryaponosomiasis, Vector, Gene flow, Census size

## Abstract

**Background:**

*Glossina fuscipes fuscipes* is the primary vector of trypanosomiasis in humans and livestock in Uganda. The Lake Victoria basin has been targeted for tsetse eradication using a rolling carpet initiative, from west to east, with four operational blocks (3 in Uganda and 1 in Kenya), under a Pan-African Tsetse and Trypanosomiasis Eradication Campaign (PATTEC). We screened tsetse flies from the three Ugandan PATTEC blocks for genetic diversity at 15 microsatellite loci from continental and offshore populations to provide empirical data to support this initiative.

**Methods:**

We collected tsetse samples from 11 sites across the Lake Victoria basin in Uganda. We performed genetic analyses on 409 of the collected tsetse flies and added data collected for 278 individuals in a previous study. The flies were screened across 15 microsatellite loci and the resulting data were used to assess the temporal stability of populations, to analyze patterns of genetic exchange and structuring, to estimate dispersal rates and evaluate the sex bias in dispersal, as well as to estimate demographic parameters (N_E_ and N_C_).

**Results:**

We found that tsetse populations in this region were stable over 4-16 generations and belong to 4 genetic clusters. Two genetic clusters (1 and 2) corresponded approximately to PATTEC blocks 1 and 2, while the other two (3 and 4) fell within PATTEC block 3. Island populations grouped into the same genetic clusters as neighboring mainland sites, suggesting presence of gene flow between these sites. There was no evidence of the stretch of water separating islands from the mainland forming a significant barrier to dispersal. Dispersal rates ranged from 2.5 km per generation in cluster 1 to 14 km per generation in clusters 3 and 4. We found evidence of male-biased dispersal. Few breeders are successfully dispersing over large distances. Effective population size estimates were low (33–310 individuals), while census size estimates ranged from 1200 (cluster 1) to 4100 (clusters 3 and 4). We present here a novel technique that adapts an existing census size estimation method to sampling without replacement, the scheme used in sampling tsetse flies.

**Conclusion:**

Our study suggests that different control strategies should be implemented for the three PATTEC blocks and that, given the high potential for re-invasion from island sites, mainland and offshore sites in each block should be targeted at the same time.

## Background

The tsetse fly (*Glossina*) is a major vector of trypanosomiasis throughout sub-Saharan Africa, causing extensive morbidity and mortality in humans and livestock [1,2]. It has been estimated that economic benefits to Africa from the eradication of tsetse could reach US$4.5 billion per year. Currently, no vaccines exist to prevent the disease and available drugs to treat HAT are expensive, can cause severe side-effects, and are difficult to administer in remote villages [3]. As a consequence, an effective alternative for controlling the disease is to target the tsetse vector [4,5]. A variety of methods to control tsetse populations are available, including habitat modification around homesteads, trapping, insecticide-treated targets, insecticide-treated cattle, and aerial or ground spraying. The release of sterile or transgenic insects has been either used or proposed as an additional control measure [4-9]. Tsetse control is implemented using two strategies: eradication and suppression. Eradication aims at eliminating fly populations from a given area, while the objective of suppression is to greatly reduce population size.

Genetic data provide a powerful tool to help identify appropriate vector control strategies, as they can be used to estimate spatial and temporal differentiation of populations, and patterns and extent of migration. These data, together with ecological and environmental, data can be used to customize vector control efforts according to local conditions and species in order to determine whether eradication or suppression is appropriate [10,11]. For example, studies of tsetse in Burkina Faso, Guinea and Senegal have identified populations that are sufficiently isolated to warrant attempts at complete eradication [10-12]. Studies elsewhere have documented relatively high levels of gene flow, necessitating integration of barriers into eradication schemes [13,14], or warranting an area-wide control effort that encompasses populations linked by gene flow [15,16]. Regional studies such as the one on *G. palpalis palpalis* in west and central Africa [17] have provided information that is useful for control efforts at a regional scale. Other population genetic studies have pointed to specific populations at a local level where control and detection methods need improvement [18].

In 2001, the Organization for African Unity (OAU) launched a new initiative, the Pan-African Tsetse and Trypanosomiasis Eradication Campaign (PATTEC) to eradicate the tsetse flies from a vast area of sub-Saharan Africa (~10 million km^2^, seven *Glossina* species) by first reducing populations using area-wide approaches (odor-baited traps, insecticide-treated targets, pour-ons and ultra-low-volume aerial spraying), followed by massive release of sterile males to ensure eradication [19]. In Uganda, phase I of this initiative has been initiated in the Lake Victoria basin, which is infested with *Glossina fuscipes fuscipes* (*Gff*). A program (Farming in Tsetse Controlled Areas, FITCA), which ended in 2004, reduced tsetse populations by 75% to 90% in the mainland sites of the target area, but the program did not include islands [20]. By 2009, a PATTEC baseline survey revealed that mainland tsetse populations had rebounded to the high levels prior to the FITCA initiative [21].

**Figure 1:**
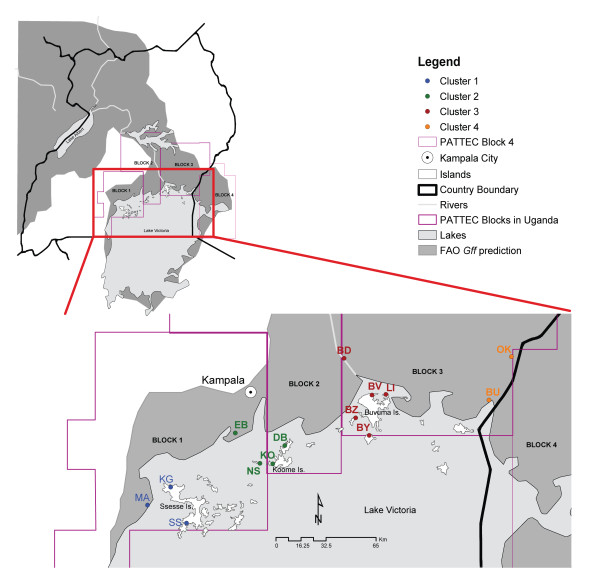
**Map of sampling sites. ** Location of sampling sites (colored dots and location codes) is shown with reference to the three intervention blocks (purple contours) defined by PATTEC in Uganda. The fourth block in Kenya is also shown. Dot and location code color denotes the genetic cluster to which the majority of individuals at each site were assigned (blue – cluster 1, green – cluster 2, red – cluster 3, and orange – cluster 4). The inset in the upper left corner shows the location of sampling sites and PATTEC blocks with reference to the whole of Uganda and neighboring countries.

The PATTEC plan, unlike FITCA, includes islands and intends to eradicate *Gff* from the Lake Victoria basin progressively from west to east [22]. The basin has been partitioned into four operational blocks (3 in Uganda and 1 in Kenya) based on the Food and Agriculture Organization (FAO) predicted habitat suitability for *Gff*, natural barriers, major urban areas, international borders and drainage patterns [22,23]. Block 1 (Figure 1), targeted for *Gff* eradication during PATTEC phase 1, is the most isolated block due to the expansion of the city of Kampala and subsequent urbanization and habitat fragmentation of the surrounding area. Block 2 has been targeted for control to create a buffer between the eradication block and the rest of the *Gff* predicted range in this area of Uganda (Figure 1). Only vector population monitoring activities are planned for the other two blocks during phase 1. Upon successful eradication in block 1, block 2 would become the eradication target and so on until the whole basin is tsetse-free. To support the PATTEC initiative, the government of Uganda and the International Atomic Energy Agency (IAEA) is planning a trial eradication of *Gff* on a remote island in the region (IAEA Project UGA5033) because of previous success of tsetse eradication on islands in Equatorial Guinea [24] and Zanzibar [25].

We used genetic variation at 15 microsatellite DNA loci to examine the genetic differentiation of *Gff* populations within and between the three Ugandan PATTEC blocks. We used these data to estimate effective population size (N_E_), to evaluate temporal stability over 4-16 generations, and to measure genetic exchange and dispersal rates within and between blocks. In view of the finding that high levels of genetic exchange occur among continental Ugandan populations of *Gff* separated by distances smaller than 100 km [16], we embarked upon an investigation of gene flow patterns at a finer scale and used the results to assess the validity of the PATTEC operational blocks with respect to vector control, to provide suggestions regarding control strategies in each block, and to evaluate the possibility of using *Gff* island populations for eradication trials.

## Methods

### Sampling

We sampled *Gff* at 3 continental localities across the Lake Victoria basin in Uganda (sites BD, EB, and MA: Table 1 and Figure 1) and at 8 localities from three groups of islands in Lake Victoria (sites BV, BY, BZ and LI from Buvuma islands; DB, NS and KO from Koome islands; and KG from Ssese islands, Table 1 and Figure 1). We used 409 tsetse flies from the above 11 sampling sites for genetic analyses. Several localities were sampled over two to three months both in the wet (April-June 2010) and the dry (February-March 2010 or February-March 2011) season (Table 1a). In addition to the data we collected from these 11 sites, we included data collected for 278 individuals sampled between the wet season of 2008 and the dry season of 2009: BU, OK [26] and SS (unpublished) (Table 1b). Assuming that *Gff* goes through approximately 8 generations per year [27,28], seasonal samples were about 4 generations apart. Tsetse flies were caught using biconical traps [29] and preserved individually in cryo-tubes containing 90% ethanol.

**Table 1:** Sample collection information and genetic summary statistics

**Population**	**Code**	**N**	**Longitude**	**Latitude**	**Season**	**Collection Date**	**Seasonal and Total**							
							**Mean AR**		**Mean H**_**O**_		**Mean H**_**E**_		**Mean F**_**IS**_	
**(a) New sites for this study**														
Budondo	BD	35	33.1209	0.5208	dry	March, 2011	4.867	4.867	0.516	0.516	0.513	0.513	−0.005	−0.005
Buvuma Is.	BV	21	33.2788	0.1368	dry	Feb, 2010	3.867	4.800	0.446	0.477	0.476	0.506	0.076	0.057
Buvuma Is.	BV	39	33.2788	0.1368	wet	June, 2010	4.400		0.493		0.513		0.035	
Bugaya Is.	BY	27	33.2684	0.0675	dry	Feb, 2010	4.067	4.867	0.499	0.486	0.496	0.497	−0.017	0.011
Bugaya Is.	BY	35	33.2684	0.0675	wet	June, 2010	4.400		0.476		0.490		0.018	
Buziri Is.	BZ	7	33.1883	0.1716	dry	Feb, 2010	2.933	3.600	0.441	0.454	0.460	0.477	0.064	0.051
Buziri Is.	BZ	11	33.1883	0.1716	wet	June, 2010	3.133		0.461		0.465		0.021	
Lingira Is.	LI	29	33.3532	0.3168	dry	Feb, 2010	4.933	5.200	0.519	0.509	0.511	0.509	−0.021	0.002
Lingira Is.	LI	17	33.3532	0.3168	wet	June, 2010	4.000		0.494		0.492		0.012	
Damba Is.	DB	32	32.7659	0.0127	wet	Apr, 2010	3.067	3.067	0.394	0.394	0.394	0.394	0.015	0.015
Koome Is.	KO	40	32.6879	−0.0911	wet	Apr, 2010	3.267	3.267	0.318	0.318	0.360	0.360	0.187	0.187
Nsazi Is.	NS	16	32.6296	−0.0956	wet	Apr, 2010	3.000	3.000	0.322	0.322	0.340	0.340	0.114	0.114
Entebbe	EB	35	32.4852	0.0823	dry	March, 2011	3.667	3.667	0.413	0.413	0.418	0.418	0.006	0.006
Kalangala Is.	KG	32	32.1083	−0.2267	wet	March, 2010	3.000	3.000	0.288	0.288	0.302	0.302	0.098	0.098
Masaka	MA	33	31.9852	−0.3564	dry	March, 2011	2.867	2.867	0.329	0.329	0.296	0.296	−0.098	−0.098
**(b) Sites from previous studies**														
Busime	BU	39	33.9711	0.2508	wet	March, 2008	4.733	5.400	0.486	0.486	0.507	0.502	0.027	
Busime	BU	40	33.9711	0.2508	wet	March, 2009	4.467		0.494		0.499		0.047	
Busime	BU	40	33.9711	0.2508	dry	Oct, 2009	4.200		0.479		0.487		0.012	
Okame	OK	39	33.3532	0.3168	wet	March, 2008	4.000	4.533	0.483	0.516	0.529	0.535	0.083	0.032
Okame	OK	40	33.3532	0.3168	wet	March, 2009	3.933		0.541		0.532		−0.010	
Okame	OK	39	33.3532	0.3168	dry	Oct, 2009	4.133		0.524		0.532		0.012	
Ssese Is.	SS	40	32.1691	−0.5022	wet	Apr, 2009	3.467	3.467	0.333	0.333	0.332	0.332	−0.016	−0.016

Summary statistics are based on 15 microsatellite loci, both for (a) recently collected tsetse specimens and (b) flies collected for previous studies. N – number of individuals analyzed. Mean across all loci of AR – allelic richness, H_O_ – observed heterozygosity, H_E_ – expected heterozygosity and F_IS_ – inbreeding coefficient. AR, H_O_, H_E_ and F_IS_ are reported for the dry and the wet season separately (seasonal), and for both seasons (total).

### Data collection

DNA was extracted from tsetse legs using the PrepGEM Insect DNA extraction kit (ZYGEM Corp Ltd, Hamilton, New Zealand) as per the protocol provided by the manufacturer. We collected genotypic data across 18 microsatellite loci (see Additional file 1: Supplementary Material for technical details), including the 13 loci used in Beadell *et al*. [16]. The other 5 (GmmA06, GmmB20, GmmD15, GmmL03, GmmL11) were selected among the loci described in Hyseni *et al*. [30]. Due to low amplification and scorability of large alleles, locus Pgp17 was excluded from all analyses. We also excluded locus GpC5b, because it was monomorphic for all populations, excepting BZ and NS (in both cases, F_IS_ = 1), and locus GmmD15, because it was monomorphic in all of the 11 sites we sampled. Thus, we only used 15 loci for all subsequent genetic analyses.

### Genetic analyses

We used *Genepop 4.1*[31] to test for deviation from Hardy-Weinberg equilibrium (HWE) and also test for linkage disequilibrium (LD). For loci with fewer than four alleles, the complete enumeration method [32] was used. All other loci were tested using the Guo and Thompson [33] Markov chain method with 100,000 dememorizations, 1,000 batches and 10,000 iterations per batch. We also used *Genepop* to carry out global tests across loci for heterozygote deficiency and heterozygote excess. Significance values were adjusted for multiple testing (HWE) and comparisons (LD) using the Benjamini-Hochberg method [34] with a false discovery rate of 0.05. Summary statistics, including allele frequencies, allelic richness, observed heterozygosity (H_O_), expected heterozygosity (H_E_) and the inbreeding coefficient (F_IS_) were calculated using the program *Genalex 6.41*[35]. In order to assess the statistical significance of genetic differentiation between temporal samples, we used *Genepop* to perform Fisher’s exact test on both microsatellite alleles and genotypes.

We used the model-based Bayesian clustering method implemented in *Structure 2.3.3*[36] to determine the genetic structure present among *Gff* populations in the Lake Victoria basin (details in Additional file 1: Supplementary Material). In addition to the Bayesian clustering implemented in *Structure*, we used a two-tiered multivariate ordination analysis, which makes no assumptions about deviations from Hardy-Weinberg and linkage equilibrium. This multivariate procedure, discriminant analysis of principal components (DAPC), has been shown to perform better than the Bayesian clustering approach when hierarchical and clinal structure is present in the data [37]. We used the *adegenet* package [38] in R [39] for the DAPC (details in Additional file 1: Supplementary Material).

In order to quantify the genetic heterogeneity of Lake Victoria populations, we computed pairwise F_ST_ values [40] among sampling localities and among genetic clusters. F_ST_ values were obtained using the program *Arlequin 3.5*[41] and their significance computed via 10,000 permutations. F_ST_ describes the genetic structure produced by non-random distribution of individuals among subpopulations relative to the total population. However, our sample may contain multiple hierarchical levels of genetic differentiation, such as genetic clusters of populations with further partitioning within clusters, including potential genetic structure arising from isolation of island from mainland populations. In order to determine the contribution of different hierarchical levels to the observed genetic structure, we estimated hierarchical F-statistics using the method described in Yang [42] (details in Additional file 1: Supplementary Material) and implemented in the R package *hierfstat*[43].

To determine whether the genetic heterogeneity of *Gff* around Lake Victoria could be attributed to differences in dispersal ability between male and female flies, we performed t-tests on pairwise relatedness between individuals within genetic groups. Relatedness was computed using maximum likelihood estimation [44] implemented in *Kingroup 2*[45]. Sex-biased dispersal was also assessed using three tests [46] implemented in *Fstat 2.9.4*[47] (details in Additional file 1: Supplementary Material).

Isolation by distance (IBD) and dispersal were evaluated using Rousset’s procedure [48] within genetic groups, using both a one-dimensional (1D) and a two-dimensional (2D) stepping-stone model. We compared the two models in order to determine differences in dispersal ability along Lake Victoria depending on whether movement happens along a line or across a surface (details in Additional file 1: Supplementary Material).

We used two methods to test for individual migrants between geographically neighboring genetic clusters (clusters 1 and 2, clusters 2 and 3, and clusters 3 and 4). In the first approach, we used the software *Geneclass 2.0*[49] to compute the likelihood of individual assignment based on regional allele frequencies [50,51]. In the second approach, we used *Flock 2.0*[52] to assign genetically similar individuals to *k* partitions (details in Additional file 1: Supplementary Material).

We estimated effective and census population sizes for each genetic group. The geographic distance between the genetic groups should reduce the bias in estimation of effective population size (N_E_) that could be introduced by migration, which influences linkage disequilibrium as well as temporal methods of N_E_ estimation [53]. N_E_ was computed using Waples and Do’s LD method implemented in the program *LDNe*[54]. We also used two temporal methods to estimate N_E_: 1) a Bayesian algorithm based on coalescence and implemented in the program *TM3*[55] and 2) a pseudo-likelihood method [56] implemented in *MLNE*. Census size (N_C_) was computed via a sequential Bayesian method [57] adapted from Gazey and Staley [58] using an R software script [59]. This method applies to a sampling scheme with replacement (non-invasive genetic sampling). Tsetse sampling, however, was done without replacement. We designed a method to account for the difference in sampling, which allowed us to utilize the adapted Gazey-Staley method [57,58] to estimate N_C_ (details in Additional file 1: Supplementary Material).

## Results

We observed the lowest H_O_ and H_E_ values in KG (0.29 and 0.30, respectively) and the highest in OK (0.54 and 0.53, respectively), while mean allelic richness across the 15 loci ranged from 2.87 in MA to 5.40 in BU (Table 1). The highest F_IS_ value was observed in KO (0.187). After applying the Benjamini-Hochberg false discovery rate procedure [34] to the multiple testing of HWE, significant deviation from HWE was only observed in KO, which was due to significant heterozygote deficit. After applying the same correction for multiple comparisons of linkage between loci, no evidence of significant LD was found.

### Temporal stability

We examined the temporal stability of the samples for which temporal collections were available. Pairwise F_ST_ values between different sampling seasons revealed temporal homogeneity, i.e. temporal samples from the same sites were not significantly genetically differentiated. The smallest difference was observed between BU seasonal samples (F_ST_ = -0.001, P = 0.63) and highest in BY (F_ST_ = 0.003, P = 0.24). This was consistent with the similarity in allele frequencies between seasons observed in Additional file 2: Figure S1. We also carried out Fisher’s exact test on microsatellite alleles and genotypes and found no significant differences between temporal samples ( Additional file 3: Table S1).

### Patterns of genetic differentiation

**Figure 2:**
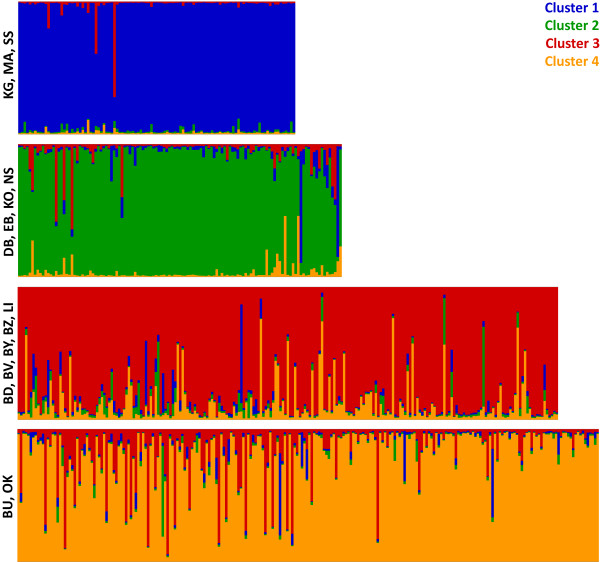
**Bayesian clustering. ** Probability of assignment of individuals from 14 sampling sites (abbreviated with two-letter codes; see Table 1) to each of the 4 identified clusters (blue – cluster 1, green – cluster 2, red – cluster 3, and orange – cluster 4) is denoted by the color composition of individual vertical bars.

**Figure 3:**
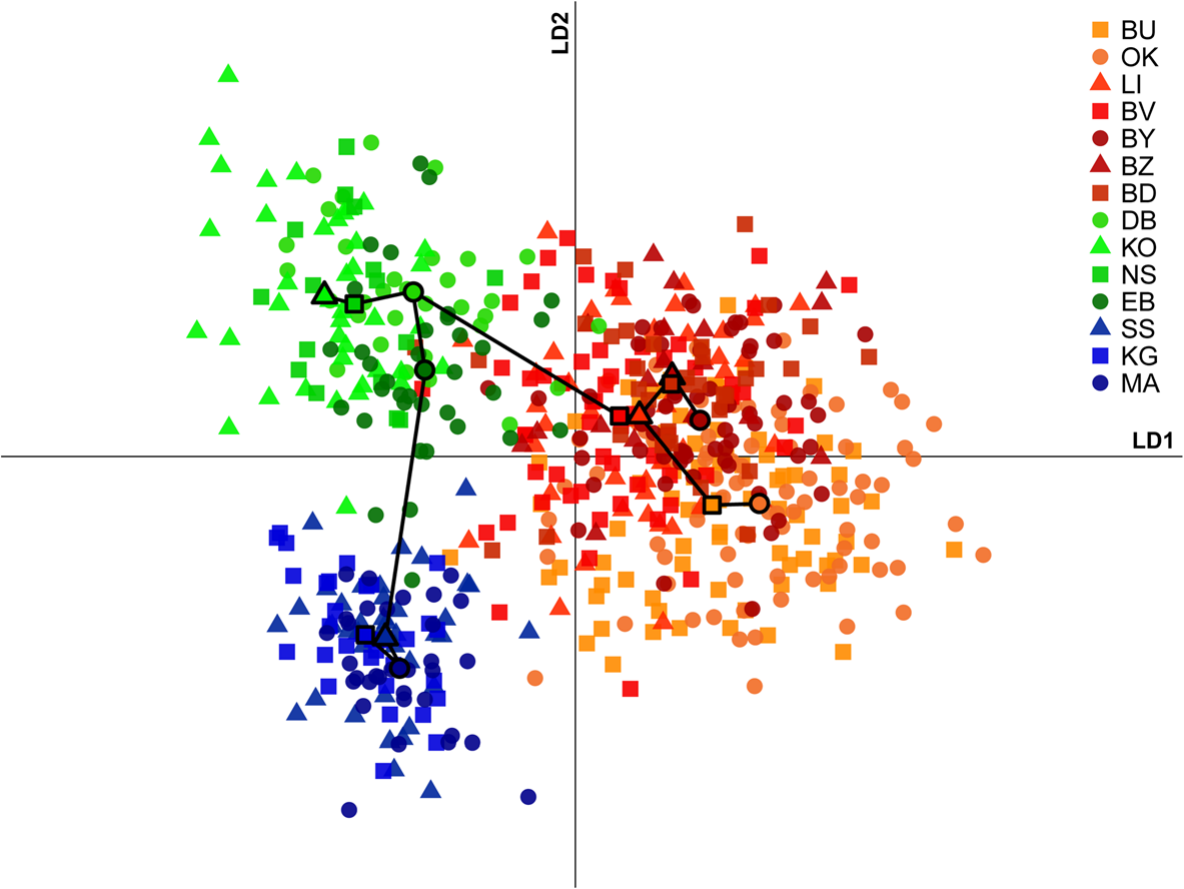
**Discriminant analysis of principal components. ** Following selection of 15 principal components using a-score optimization, two linear discriminants (LD1 and LD2) were used to plot tsetse individuals represented in orange (cluster 4), red (cluster 3), green (cluster 2) and blue (cluster 1). Also shown is a minimum spanning tree between the centroids of the 14 populations.

Using the Evanno criterion of ΔK, the results of the Bayesian analysis in *Structure* identified four distinct genetic clusters (Figure 2). The DAPC approach (Figure 3), concordantly, detected four clusters comprising the same populations. Clusters 1 and 2 are within PATTEC blocks 1 and 2. However, one continental site and one island site within block 1 (EB and NS) are genetically closer to the continental and island sites in cluster 2 than the other samples from block 1. Cluster 3 includes the Buvuma islands and adjacent mainland sites and cluster 4 includes two inland sites to the east (BU and OK). Both clusters are within PATTEC block 3. While the two westernmost clusters (1 and 2) are genetically distinct, from each other (F_ST_ = 0.184, Additional file 4: Table S2) and from clusters 3 and 4 (F_ST_ = 0.124-0.191, Additional file 4: Table S2), there is a great amount of gene flow between clusters 3 and 4. The F_ST_ value between clusters 3 and 4 (F_ST_ =0.036), while significantly different, is much lower than among other clusters. The extent of gene flow between clusters 3 and 4 is also evident from posterior probabilities of assignment obtained from *Structure* (Figure 2) as well as from the DAPC method (Figure 3; posterior probabilities not shown).

The analysis of hierarchical F-statistics (Table 2) revealed only one significant hierarchical level of genetic structure, i.e. the subdivision into three genetic groups (group 1 = cluster 1, group 2 = cluster 2, group 3 = clusters 3 and 4; F_3/T_ = 0.114, P = 0.001; Table 2a). Subdivision of group 3 into clusters 3 and 4 did not contribute significantly to the observed genetic structure (F_4/3_ = 0.019, P = 0.072; Table 2a). Island areas with nearby mainland sites were also analyzed separately in order to look at the contribution of distance between mainland and island sites to the genetic structuring of each cluster. Tsetse flies from island sites were not significantly isolated from mainland flies within the same cluster (cluster 1: F_IM/T_ = −0.017, P = 1.000; cluster 2: F_IM/T_ = 0.042, P = 0.259; cluster 3: F_IM/T_ = 0.009, P = 0.204; Table 2b-d). Consistent with this finding, pairwise F_ST_ values ( Additional file 5: Table S3) showed that absence of significant differentiation does not carry the proviso that the compared sites be situated on the same island or that the comparison not be between island and mainland sites. For instance, in group 1, the island site, SS, was not significantly different from the mainland site, MA (F_ST_ = 0.007; Additional file 5: Table S3), but it was significantly different from the other island site, KG (F_ST_ = 0.024; Additional file 5: Table S3).

**Table 2:** Hierarchical F-statistics

**(a) Entire sample**				
	**3 Regions**	**4 Clusters**	**Populations**	**Individuals**
**Total**	0.114 (F_3/T_)	0.130 (F_4/T_)	0.151 (F_Pop/T_)	0.173
**3 Regions**		0.019 (F_4/3_)	0.043 (F_Pop/3_)	0.067
**4 Clusters**			0.024 (F_Pop/4_)	0.049
**Populations**				0.026
**(b) Cluster 1**				
		**Island/Mainland**	**Populations**	**Individuals**
**Total**		−0.017 (F_IM/T_)	0.009 (F_Pop/T_)	0.001
**Island/Mainland**			0.025 (F_Pop/IM_)	0.017
**Populations**				−0.009
**(c) Cluster 2**				
		**Island/Mainland**	**Populations**	**Individuals**
**Total**		0.042 (F_IM/T_)	0.071 (F_Pop/T_)	0.123
**Island/Mainland**			0.030 (F_Pop/IM_)	0.084
**Populations**				0.055
**(d) Cluster 3**				
		**Island/Mainland**	**Populations**	**Individuals**
**Total**		0.009 (F_IM/T_)	0.021 (F_Pop/T_)	0.048
**Island/Mainland**			0.012 (F_Pop/IM_)	0.039
**Populations**				0.028

***(a)*** Hierarchical F-statistics computations were carried out for the entire sample based on 3 hierarchical levels: (1) populations (sampling sites), (2) populations grouped into four clusters, and (3) clusters 3 and 4 grouped together. ***(b)-(d)*** Hierarchical F-statistics were computed separately for clusters 1–3 (containing populations from Ssese, Koome and Buvuma islands, respectively), based on 2 levels: (1) populations, and (2) populations grouped into island and mainland.

**Figure 4:**
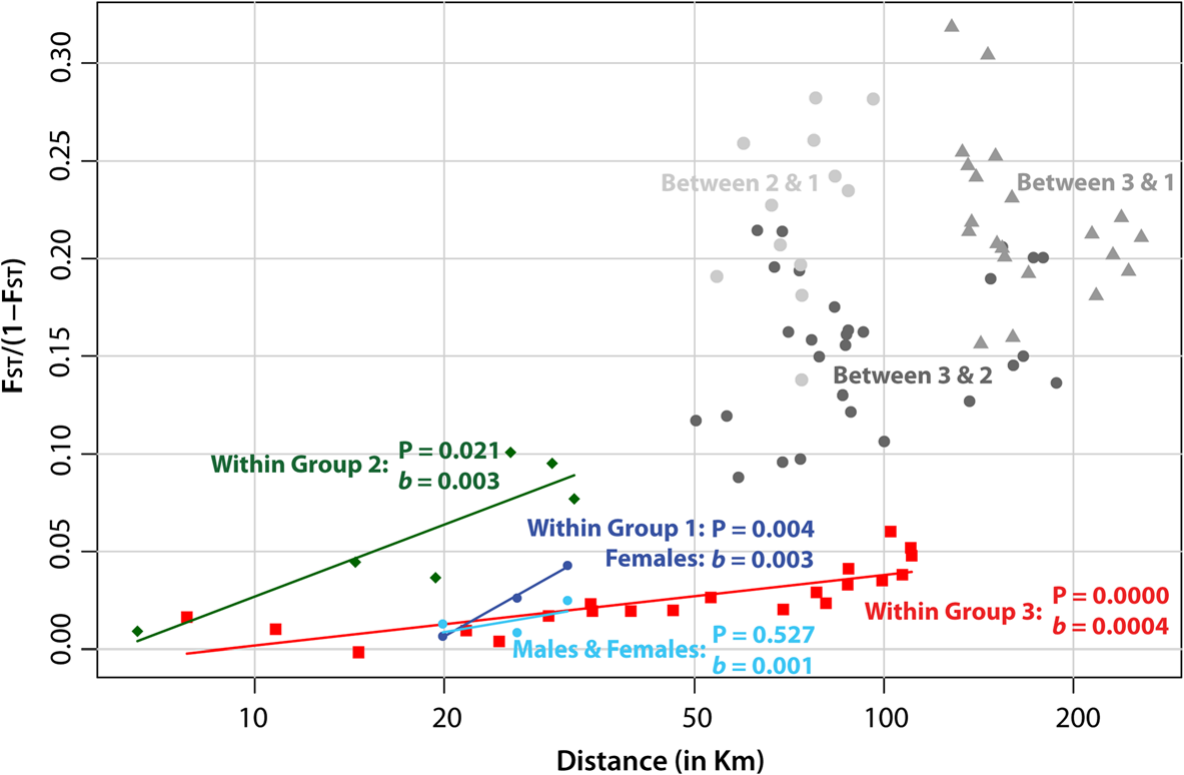
**Isolation by distance. ** Isolation by distance between sites within clusters 1, 2 and the group of clusters 3 and 4. Linearized F_ST_ (i.e., F_ST_/(1-F_ST_)) values were regressed against geographic distance (in kilometers). Slopes (*b*) and P-values are shown. For cluster 1, two isolation-by-distance models are pictured (light blue = both male and female individuals; dark blue = females only). In addition to populations within clusters (blue = cluster 1, green = cluster 2, red = clusters 3 and 4), populations belonging to different clusters were regressed, separately, and are depicted in three shades of grey.

Having delineated three genetic groups through multiple methods, we regressed linearized F_ST_ (i.e., F_ST_/(1-F_ST_)) values against geographic distance to evaluate the occurrence of isolation by distance (IBD) within these groups (Figure 4). We detected significant IBD within groups 2 and 3 (1D: P = 0.021, P = 0.000; 2D: P = 0.022, P = 0.000). IBD within group 1 was not significant when males were included in the model (1D: P = 0.527; 2D: P = 0.568). We did, however, find significant isolation by distance among females in group 1 (1D: P = 0.004; 2D: P = 0.044). In addition to the local patterns within groups, global linearized F_ST_ patterns between groups revealed that group 1 is as isolated from the more adjacent group 2 as it is from group 3 (Figure 4), which reiterated the DAPC results (Figure 3).

### Dispersal and migration

We tested for sex-biased dispersal within the three genetic groups using four methods (Table 3). Three of the four methods showed evidence of male-biased sex dispersal. The F_ST_-based method did not reveal significant differences between sexes, possibly because of the reduced power of this method for low dispersal rates [46]. In group 3, male-biased dispersal was only supported by mAIc (P = 0.012, Table 3). Evidence for male-biased dispersal was stronger in group 1 (vAIc and mPr were significantly different between males and females) and group 2 (mAIc, vAIc and mPr were significant).

**Table 3:** Sex-biased dispersal

		**F**_**ST**_	**mAI**_**C**_	**vAI**_**C**_	**mPr**
**Clusters 3 & 4**	**F (202)**	0.028	0.476	20.05	0.031
	**M (179)**	0.028	−0.537	17.20	0.033
	***P-value***	0.991	**0.012**	0.261	0.255
**Cluster 2**	**F (71)**	0.061	0.706	11.64	0.115
	**M (52)**	0.076	−0.964	22.15	0.137
	***P-value***	0.617	**0.024**	**0.025**	**0.001**
**Cluster 1**	**F (61)**	0.020	0.541	17.19	0.099
	**M (44)**	0.013	−0.750	35.52	0.252
	***P-value***	0.931	0.144	**0.033**	**0.000**

Female and male dispersal were compared within three groups: (1) cluster 1, (2) cluster 2, and (3) clusters 3 and 4. Sex differences in dispersal were evaluated using four different statistics, F_ST_, mAIc, vAIc (mean and variance, respectively, of the Favre *et al*. [60] assignment index) and mPr (mean pairwise relatedness (based on Konovalov and Heg [44]). P-values in bold type are significant.

We calculated per generation dispersal (σ), dispersal surface (σ^2^), Wright’s neighborhood size (W_N_) and migration rate (*m*), using both 1D and 2D stepping-stone models (Table 4). In groups 1 and 2, dispersal distance σ (group 1 (females): 1D = 3.9 km, 2D = 2.5 km; group 2: 1D = 4.5 km, 2D = 3.6 km), dispersal surface σ^2^ (2D: 6.2 km^2^ and 12.9 km^2^), and neighborhood size (13 and 19 individuals) were similar to each other. These estimates were higher for group 3 samples (2D: σ^2^ = 200.1 km^2^, W_N_ = 64 and σ = 14.1 km). Migration rate estimates per generation were also higher in group 3 (0.033) than the other two genetic groups (0.013 in group 1 (females) and 0.025 in group 2).

**Table 4:** Population size, migration and dispersal estimates

	**Linkage Diseq.**		**Temporal (*MLNE*)**		**Temporal (*TM3*)**		**Mean N**_**C**_	***95% HPD***
	**(*LDNe*)**		**(Likelihood)**		**(Bayesian)**			
	**Mean N**_**E**_	***(95% CI)***	**Mean N**_**E**_	***(95% CI)***	**Mean N**_**E**_	***(95% CI)***		
Clusters 3 & 4	310	(258–380)	243	(181–327)	240	(223–250)	4121	(2422–6109)
Cluster 2	121	(88–180)	45	(35–61)	33	(22–47)	1199	(470–2191)
Cluster 1	157	(98–326)	212	(106–611)	178	(105–285)	1299	(412–2621)
	**N**_**E**_**/N**_**C**_	**σ (km)**	**σ (km)**	**σ**^**2**^**(km**^**2**^**)**	***m***	**W**_**N**_		
		**(1D)**	**(2D)**	**(2D)**	**(2D)**	**(2D)**		
	**Based on LDNe estimates**							
Clusters 3 & 4	0.075	15.16	14.14	200.06	0.033	64		
Cluster 2	0.101	4.53	3.59	12.92	0.025	19		
Cluster 1	0.121	*3.94*	*2.49*	*6.21*	*0.013*	*13*		

Estimates were computed for clusters 1, 2 and the group comprising clusters 3 and 4. Effective population size (N_E_) was computed using both a linkage disequilibrium method (in *LDNe*) and two temporal methods (a likelihood approach implemented in *MLNE* and a Bayesian approach implemented in *TM3*). Mean N_E_ and parametric 95% confidence intervals are shown. Census size (N_C_) was computed using a sequential Bayesian method [51]. Mean N_C_ and 95% highest probability density (HPD) intervals are shown. Dispersal distance (σ) was estimated using both a one-dimensional (1D) (F_ST_/(1-F_ST_) ~ *a* + *b*GD; GD = geographic distance in kilometers) and a two-dimensional (2D) (F_ST_/(1-F_ST_) ~ *a* + *b*ln(GD); ln(GD) = log of geographic distance) isolation-by-distance model. Dispersal surface (σ^2^), migration (*m*) and Wright’s neighborhood size (W_N_) estimates from the 2D model are also reported. σ, σ^2^, *m* and W_N_ in cluster 1 were only computed for female individuals (the model was not significant when males were considered) and are shown in italic type.

**Figure 5:**
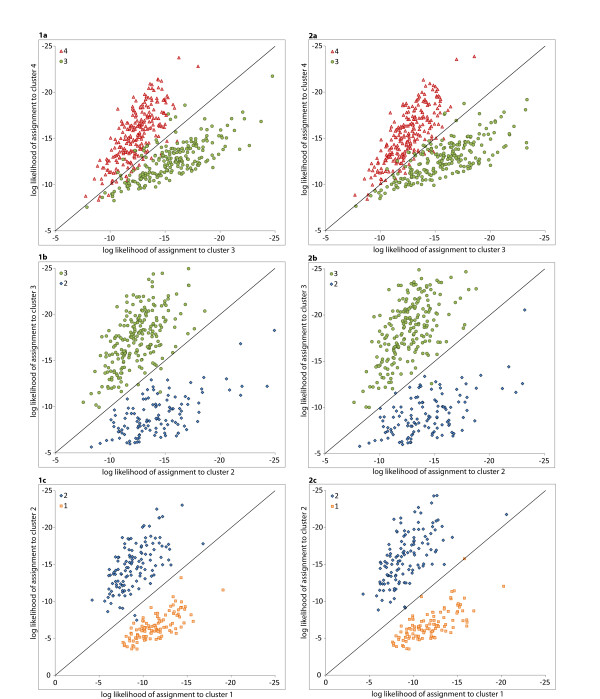
**Migrants. ** Scatterplots of log-likelihood of assignment of individuals to the cluster of origin and neighboring clusters. Each dot represents an individual and its color denotes the individual’s cluster of origin (red = cluster 4, green = cluster 3, blue = cluster 2 and orange = cluster 1; plots 1a-1c: *Flock* results, and 2a-2c: *Geneclass* results). Diagonal lines represent a log-likelihood ratio of 0. Migrants are represented by dots found across diagonals, away from their cluster of origin.

Figure 5 shows the scatterplots of log-likelihood of assignment of individuals to their cluster of origin and neighboring clusters using *Flock* and *Geneclass*. Migrant detection using these two methods was largely congruent with assignment of individuals to clusters using *Structure* (Figure 2) and DAPC (Figure 3; posterior probabilities not shown). Using log-likelihood ratios <0.5, we identified two migrants from cluster 1 in cluster 2 and four migrants from cluster 2 in clusters 3 and 4. The genetic exchange between clusters 3 and 4 was much higher, with 14 migrants from Buvuma islands in the BU-OK region and 12 individuals who migrated in the other direction. These two clusters shared 26 to 56 migrants for log-likelihood ratios <0.5 to <1.0, respectively.

### Population size

Estimates of population size were computed for each genetic group. Table 4 shows effective population size (N_E_) estimates computed using both a linkage disequilibrium method (*LDNe*) and two temporal methods (a likelihood approach implemented in *MLNE* and a Bayesian approach implemented in *TM3*). Table 4 also reports the estimated census size (N_C_).

N_E_ and N_C_ were estimated for a dataset consisting of 12, 4, and 16 generations of flies from groups 1, 2 and 3, respectively. For the temporal methods only the first and the last samplings were used for N_E_ estimation, as the increased sampling interval might decrease the bias caused by overlapping generations and age structure [53]. With the exception of flies in group 1, the estimates obtained with the *LDNe* and temporal methods were very similar (Table 4). The lower temporal estimates for group 1 could be due to the higher substructure (F_ST_ between EB and other populations in the region was 0.07-0.09). We used N_E_ estimates obtained via the LD method to calculate dispersal. N_C_ was much higher in group 3 (4,121; Table 4) than elsewhere (1,199 in group 2 and 1,299 in group 1). The N_E_/N_C_ ratio was lowest (0.075) in group 3; this ratio was higher in group 2 (0.101) and highest in group 1 (0.121).

## Discussion

### Temporal stability

The genetic data we collected for 14 sampling sites (Table 1 and Figure 1), which span up to 16 generations, showed that *Gff* populations are genetically stable over multiple seasons. Recent work on mainland sites in Uganda on the same species corroborates this result [26]. The significant genetic non-differentiation over several generations seems counterintuitive, given seasonal fluctuations in *Gff* abundance [61,62]. If this fluctuation is due to actual population reduction, however, 16 generations might not be enough time for genetic drift to produce differentiation, even at low N_E_ values (33–310 individuals), especially if migration alleviates the effects of drift. Seasonal fluctuations in population size could also be a reflection of the low efficiency of trapping devices used to monitor fly populations [63]. *Gff* is susceptible to high temperatures [64] and is known to inhabit the most humid habitats [65,66]. The reduced fly catches during the dry season could thus be due to refuge-seeking behavior whereby flies hide under bushes that cannot be easily accessed by traps. This behavior is shared by other riverine tsetse species [67,68]. Reduced movement during the dry season has been reported for *G. tachinoides* in northern Nigeria, which, like *Gff*, belongs to the *palpalis* group [69].

At the offshore island sites BY, BZ, and LI (Table 1), where temperatures are constantly lower and humidity is relatively high due to high rainfall and high evaporation rates throughout the year [70], dry and wet season fly catches are comparable in size. An exception to this is the low dry season tsetse abundance on the Buvuma islands (BV; Table 1). Strong dry-season winds, which are known to impact *Gff* activity [71,72] and commonly occur on Buvuma, might be responsible for the low fly densities recorded there. Windy conditions have also been reported to affect tsetse movement in Nigeria [73]. Additionally, given the genetic similarity between flies on the Buvuma islands and flies found in BY, BZ and LI, it is likely that Buvuma flies seek respite in adjacent habitats from harsh local dry season conditions.

### Patterns of genetic differentiation and dispersal within and between clusters

Clustering (Figure 2) and multivariate (Figure 3) analyses detected four genetic clusters of tsetse populations in the Ugandan Lake Victoria basin. Based on DAPC (Figure 3), F_ST_ ( Additional file 4: Table S2), hierarchical F-statistics (Table 2) and IBD analyses (Figure 4), levels of genetic differentiation varied among clusters; while flies from clusters 1 and 2 were genetically distinct from each other and flies from clusters 3 or 4, the latter two clusters exchanged a large amount of genetic information. This was also confirmed by individual likelihood assignment tests, which identified numerous migrants (26 to 56) between clusters 3 and 4 (Figure 5). Clusters 1 and 2 (i.e., groups 1 and 2) correspond approximately to blocks 1 and 2, respectively, which were identified by PATTEC for eradication and suppression. However, two sites in block 1 are genetically closer to sites from block 2 (Figure 1). Clusters 3 and 4 (i.e., group 3) fall within block 3 targeted for initial monitoring in the first phase of the rolling carpet initiative.

The high level of genetic connectivity between the two easternmost clusters (clusters 3 and 4) could be facilitated by the presence of suitable *Gff* habitat, i.e., riparian and lacustrine thickets, tree canopies with understory vegetation, or patches of banana and *Lantana camara*[74-76]. These thickets have been greatly reduced through human settlement and other human activities in the areas that make up clusters 1 and 2. The higher level of isolation of tsetse flies observed in cluster 1 could be a function of increased habitat fragmentation and the intensive tsetse control activities dating back to the 1960s. In this area, high levels of deforestation have left only small gallery forests along the lakeshore. The growth of the city of Kampala could be an additional physical barrier to gene flow between flies from clusters 1 and 2. Increasing habitat fragmentation caused by human encroachment has been reported to have a major impact on the distribution, densities and structuring of riverine tsetse species [13,77,78].

Within clusters, island populations were not genetically differentiated from the mainland ones (Table 2 and Additional file 3: Table S1). Thus, the water body separating island and mainland sites (cluster 1: 19.9 km; cluster 2: 25.5 km; cluster 3: 34.4 km) does not act as a barrier to gene flow. Frequent human movements between islands and the mainland (fishing boats and daily ferry transport) may contribute to passive dispersal [79]. The extent of genetic connectivity of fly populations in this study is congruent with the general finding of other genetic studies on *Gff*[16,26] and other riverine species of tsetse [13,14,17,80]. In *G. p. gambiensis*, however, Solano *et al.*[81] report very low levels of migration between coastal sites and the Loos islands, situated 30 km off the coast of Guinea. Although this distance is not considerably greater than the distance separating adjacent island and mainland sites in our study (19.9-34.4 km), the differences in gene flow may be attributable to differences in the intensity of boat traffic, and thus more opportunity for passive dispersal of tsetse in the Lake Victoria region than off the coast of Guinea.

### Population size and dispersal

Population size estimates were calculated for three groups, which we defined as genetically distinct units using multiple analyses. The effective population size estimates (N_E_) were quite concordant among the linkage and temporal methods, ranging from 33 to 121 in group 2, 157 to 212 in group 1 and 240 to 310 in group 3. The N_C_ estimated for group 3 was much higher than the N_C_ estimates for groups 1 and 2. Despite the large difference in N_C_ estimates, the N_E_ estimates were similarly low in all three groups. The N_E_/N_C_ ratio was, therefore, lowest in group 3 (Table 4). This ratio is affected by family-correlated survival (i.e. how families survive as a unit [82,83]) and variance in family size at high N_C_ values [83,84]. In terms of control, this implies that reducing population size alone without eradication would not constitute a permanent solution because N_E_ levels could be maintained, or even increased when N_C_ is reduced because of a decrease in variance of family size and increase in survival of families as units. The robustness of N_E_ in the face of population reduction can be seen in group 1, which has been subjected to intensive control efforts in the past; despite the low N_C_, N_E_ was relatively high (the N_E_/N_C_ ratio was as high as 0.121 compared to 0.101 in group 2 and 0.075 in group 3).

Regression of linearized F_ST_ against geographic distance revealed significant IBD within groups 2 and 3, as well as females in group 1. Group 1 comprised only three sampling sites and, thus, we only had three data points for the regression. Despite the scarcity of data points, we observed a significant IBD pattern for the less mobile females by removing the better-dispersing male individuals from the model. Dispersal distances (σ) were similar between the 1D and 2D IBD models. These distances were approximately 14–15 km for flies in group 3 and lower in the other two groups, ranging from 2.5 to 4.5 km (Table 4). Based on the 2D estimates, the dispersal surfaces (σ^2^) within groups 1, 2 and 3 were 6.2, 12.9 and 200.1 km^2^, respectively. Similarly, migration rates per generation (*m*) within the three units ranged from 0.01 in group 1 to 0.03 in group 3. Flies in group 3 cover greater distances and disperse over a wider surface area per generation, suggesting that conditions for movement are more favorable in this region, possibly due to environmental factors.

These genetically derived dispersal distances and migration rates are very similar to estimates of dispersal rates for *Gff* based on mark-release-recapture (MRR) studies, which are about 14.2 km per generation given the movement estimate of 338 m/day [85]. Our genetic estimates are also similar to MRR estimates for other riverine species, such as *G. palpalis gambiensis* and *G. tachinoides*[13,86]. MRR data do not necessarily correlate with genetic data, as was observed for flies from the *morsitans* group [87], suggesting that although habitat fragmentation reduces dispersal capacity, it may not impact levels of intraspecific genetic cohesiveness and that its effect is species-dependent. The relatively low dispersal rates in groups 1 and 2, as compared to group 3 and MRR estimates for *Gff* suggest that the dispersal capacity of flies is reduced in these areas, probably due to habitat loss and control efforts. The reduction in dispersal capacity has had an effect on genetic cohesiveness in the region and led to the differentiation into distinct genetic groups.

Suppression should be followed by eradication in all three groups. While eradication would be harder to implement in group 3 because of the comparatively larger population size, larger dispersal distance and surface per generation, even the best suppression efforts would be much more difficult to maintain over time. Thus, the better option would be for an eradication campaign to follow an initial suppression phase post-haste, much more rapidly in group 3 than the other two groups.

The dispersal of flies across our study area showed evidence of sex bias. While male-biased dispersal in group 3 was only supported by mAIc, evidence for male-biased dispersal was stronger in groups 1 and 2. The finding of male-biased dispersal is very valuable for sterile insect technique (SIT) vector control efforts. The release of sterile male flies, as the better dispersing sex, holds the promise of sterile individuals being able to efficiently compete with wild breeders.

## Conclusion

The findings of this study have reaffirmed the importance of gathering genetic data prior to implementing area-wide tsetse vector control operations. The high levels of genetic mixing between islands and mainland sites suggest that these sites should be treated at the same time. The boundaries of the PATTEC blocks need to be modified to reflect the genetic composition of fly populations (i.e., three genetic groups). Fly populations from the two westernmost groups (1 and 2) are relatively isolated from flies in group 3, suggesting that suppression followed by eradication measures can be effective in these regions, but only if area-wide approaches include both island and mainland sites. For group 3, high levels of gene flow, which translate to a large dispersal surface (σ^2^ = 200 km^2^), as well as a very large census size and the potential for the N_E_/N_C_ ratio to increase in the case of population size reduction, all suggest that suppression alone is not likely to produce desirable results in the long run and that it should be coupled with eradication. Additionally, following the initial suppression phase, eradication would have to ensue faster for group 3 than the other two groups in order to overcome the comparatively higher dispersal capacity of flies and prevent re-infestation. The high dispersal rate in group 3 is a strong argument against the Buvuma islands being a suitable location to evaluate eradication protocols, and that the Ssese islands are a better target for this purpose, provided mainland sites are also included. The finding of male-biased dispersal of tsetse populations in this region ensures that eradication efforts involving SIT are likely to be successful.

## Competing interests

The authors declare that they have no competing interests.

## Authors’ contributions

SA, AC, LMO, CM and JOO designed the study. ABK and LMO collected samples in Uganda. CH and ABK performed the lab work. CH carried out the statistical analyses. ABK provided background and ecological information. CH and ABK wrote the initial draft of the manuscript. CH, AC, SA, and CM revised the manuscript. SA and AC jointly supervised the work at Yale University. All authors read and approved the final manuscript.

## Supplementary Material

Additional file 1 **Supplementary Material **[88,89]. Click here for file

Additional file 2 **Figure S1. ***Temporal allele frequencies. * Frequencies of alleles (above 0.05) across 15 loci are shown for the group of BU and OK samples (wet season of 2008 – dark blue, wet season of 2009 – blue, dry season of 2009 – light blue), and BV, BY, BZ and LI samples grouped together (dry season of 2010 – dark red, wet season of 2010 – light red). Click here for file

Additional file 3 **Table S1. ***Fisher’s exact differentiation test *. For each site or group of sites (BU-OK and BV-BY-BZ-LI), tsetse collected at different sampling times (wet and dry seasons; see Table 1) were tested for differences in allelic frequencies (genic differentiation) and genotypic frequencies. P (genic) and P (genotypic) – probability of non-differentiation. Click here for file

Additional file 4 **Table S2. ***Pairwise FST values. * FST values were computed for 4 clusters, averaged across 15 loci based on Weir and Cockerham [40]. FST values are reported in the lower diagonal and significance (α = 0.05) in the upper diagonal (‘-’: not significant; ‘+’: significant). Click here for file

Additional file 5 **Table S3. ***Pairwise FST values. * FST values were computed for 14 sampling sites, averaged across 15 loci based on Weir and Cockerham [40]. FST values are reported in the lower diagonal and significance (α = 0.05) in the upper diagonal (‘-’: not significant; ‘+’: significant). Non-significant FST values are represented in bold italic type. Click here for file
