# Supplementary material for: The population structure of Glossina fuscipes fuscipes in the Lake Victoria basin in Uganda: implications for vector control
Source: Parasit Vectors. 2012 Oct 4;5:222. doi: 10.1186/1756-3305-5-222 (PMC3522534; doi:10.1186/1756-3305-5-222)
Supplement: Additional file 1 — Supplementary Material [88,89]. [file 1756-3305-5-222-S1.docx]

**SUPPLEMENTARY MATERIAL**

***Microsatellite amplification and scoring***

We prepared 13 µl reactions using 1-10 ng of template DNA, 1x PCR buffer, 27.5 nmol of MgCl_2_, 2.75 nmol of each dNTP, 1 µg of BSA (New England Biolabs, Ipswich, MA, USA) and 0.5 units of GoTaq DNA polymerase (Promega Corp., Madison, WI, USA) (for loci C07, GpC5b and GmL11, 0.5 units of Taq Gold polymerase (Applied Biosystems, Foster City, CA, USA) were used). Ten loci (A03b, B05, D05, D101, GmmA06, GmmB20, GmmD15, GmmL03, GmmL11 and GpCAG133) were amplified using a fluorescently labeled forward primer and an unlabeled reverse primer. The other eight loci (C07, Gmm8, GmsCAG29B, GpB20b, GpC5b, GpC10b, Pgp17 and Pgp28) were amplified using the M13-tailed primer method [87], i.e. the forward primer is 5’-tailed with a 15-mer M13 sequence (5’-TCCCAGTCACGACGT-3’) and, in addition to the unmodified reverse primer, a third primer is included with a fluorescent dye attached to the above M13 sequence. For the labeled forward primer method, 5 pmols each of labeled forward and unlabeled reverse primer were added to the reaction, while in the M13 method, we used only 0.4 pmols of the M13-tailed forward primer and 5 pmols each of the fluorescently labeled M13 primer and unlabeled reverse primer. Amplification was carried out via touchdown PCR: after the initial denaturation (95^o^C for 5 min), reactions cycled through 95^o^C for 30s, 60^o^C to 51^o^C (1^o^C decrement/cycle) for 25s and 72^o^C for 30s in the first step, going through an additional 40 cycles of 95^o^C, 50^o^C and 72°C in the second step, concluded by a 20-minute extension at 72°C. PCR products were genotyped on an AB3730xl DNA Analyzer (Applied Biosystems) at the Yale DNA Analysis Facility on Science Hill (DAFSH) (<http://dna-analysis.research.yale.edu>). Alleles were scored using the program *Genemarker 1.95* (SoftGenetics, State College, PA, USA).

***Genetic analyses***

We used a Bayesian clustering method implemented in *Structure 2.3.3* [36] that uses a Markov chain Monte Carlo (MCMC) procedure to group individuals into genetic clusters (K) so as to minimize deviation from HWE and reduce LD. Without any prior location information, and incorporating admixture and correlated allele frequencies into the model, we performed 3 independent runs for each K between 1 and 10 using a burn-in period of 50,000 followed by 500,000 MCMC steps. We inferred the most probable K both following the suggestions of Pritchard et al. [36] and using the ∆K (second order rate of change) method [88].

To supplement the Bayesian clustering analysis, we carried out discriminant analysis of principal components (DAPC), since it tends to perform better when hierarchical and clinal structure is present [37]. DAPC comprises two steps: 1) the principal component analysis step, where we reduced the dimensionality of the multilocus allelic data by selecting the optimal 15 principal components based on a-scores (i.e., reassignment probability to the true cluster minus reassignment probability to randomly permuted clusters), and 2) the discriminant analysis step, where we used two discriminants to identify the linear combination of principal components from the first step that best distinguished prior groupings (populations) of individuals.

We computed hierarchical F-statistics using the method described in Yang [42] to assess the contribution of different levels to the observed genetic structure. The method was implemented in the R package *hierfstat* [43]. F_IM/4_ measures the homozygosity due to separation into island and mainland sites within four genetic clusters and was tested by randomizing populations between island/mainland sites within these clusters. F_4/3_ captures the homozygosity generated by subdivision within three genetic groups (group 1 = cluster 1, group 2 = cluster 2, group 3 = clusters 3 and 4) and was tested by permuting populations between the four clusters within the three groups. F_3/T_ represents the homozygosity due to separation into the three genetic groups and was measured by randomizing populations between these groups. We also estimated hierarchical F-statistics for each group of islands (Buvuma, Koome and Ssese) and their neighboring mainland sites. F_IM/T_ represents the homozygosity arising from separation between island and mainland populations and was tested by randomizing populations between island and mainland sites. For each of the above tests 10,000 permutations were carried out.

To check whether the genetic heterogeneity of *Gff* around Lake Victoria could be attributed to differences in dispersal ability between male and female flies we used relatedness analyses computed using maximum likelihood estimation [44] in the program *Kingroup 2* [45]. We corrected for bias due to population differences in allele frequencies. Significance of differences between the two sexes was determined via two-sided t-tests in R. We also used F_ST_ analyses and implemented three tests available in *Fstat 2.9.4* [47] to check for sex-biased dispersal: 1) sex-specific F_ST_, 2) the mean of the corrected assignment index (mAIc) [89], and 3) the variance of the corrected assignment index (vAIc) [89]. All three tests were two-sided and were done using 10,000 permutations. F_ST_ and mAIc are expected to be smaller and vAIc is expected to be higher in the sex with a higher dispersal rate.

To explore isolation by distance (IBD) and dispersal within each genetic group we used Rousset’s procedure [48] implementing both a one-dimensional (F_ST_/(1-F_ST_)~*a*+*b*GD) and a two-dimensional (F_ST_/(1-F_ST_)~*a*+*b*ln(GD)) stepping-stone model, where a linearized measure of differentiation, F_ST_/(1-F_ST_), is regressed on geographic distance (GD) and the natural logarithm of geographic distance, respectively (*b* is the slope of the regression). In the two-dimensional model, using the estimates of N_E_ and equations for the density of reproducing adults D (D=N_E_/ε^2^; ε - maximum geographic distance between neighboring populations within a region) and neighborhood size (4πDσ^2^=1/*b*), we calculated dispersal distance per generation σ and the second moment of dispersal distance (i.e., dispersal surface) σ^2^. We also computed migration rate using the relationship N_E_m = 2Dσ^2^. In one dimension, dispersal distance σ and migration rate were determined using one-dimensional relationships for density D= N_E_/ε, neighborhood size 4Dσ^2^=1/*b* and N_E_mε=Dσ^2^. Both regressions and subsequent calculations were carried out in the R software.

To identify individual migrants, we used two approaches. We used regional allele frequencies in a likelihood-based assignment test [50] implemented in the software *Geneclass 2.0* [49]. We used the Paetkau et al. [51] simulation routine to simulate a set of 10,000 genotypes generated using allele frequencies from the 4 clusters. To distinguish true from statistical migrants (type I error), we compared sampled genotypes to simulated genotypes and determined the signiﬁcance of assignment (α = 0.01). In the second approach, we used *Flock 2.0* [52] to assign genetically similar individuals to *k* partitions using a positive feedback mechanism where the number of individuals assigned to each of *k* groups grows with each re-allocation. We carried out a total of 50 re-allocations and performed 100 runs for *k* = 4. In order to identify true migrants, we used a log-likelihood ratio of either 0.5 or 1.

We estimated effective and census population sizes for each genetic group. We estimated effective population size (N_E_) using Waples and Do’s LD method implemented in the program *LDNe* [54], as well as two temporal methods: 1) a Bayesian algorithm based on coalescence and implemented in the program *TM3* [55] and 2) a pseudo-likelihood method [56] implemented in *MLNE*. For the LD method, we ignored alleles below a frequency of 0.5%. Census size (N_C_) was computed via a sequential Bayesian method [57] adapted from Gazey and Staley [58] using an R software script [59]. This method applies to a sampling scheme with replacement (non-invasive genetic sampling), but tsetse are sampled without replacement. We, therefore, designed a method that allows the Gazey-Staley Bayesian estimation [57, 58] of N_C_ to be applied to sampling without replacement. We multiplied the maximum of locus-specific ratios of expected-to-observed genotypes (expected genotypes = diploid combinations of all alleles in a locus) with the estimated N_E_ to obtain a preliminary N_C_. We carried out resampling, with 100,000 replications, of the preliminary population of size N_C_, using a sample size equal to the number of individuals we analyzed. The values for the number of uniquely sampled individuals and the number of total samples were then used in the sequential Bayesian method [57, 59] to derive N_C_ estimates.
