# Supplementary material for: The population structure of Glossina fuscipes fuscipes in the Lake Victoria basin in Uganda: implications for vector control
Source: Parasit Vectors. 2012 Oct 4;5:222. doi: 10.1186/1756-3305-5-222 (PMC3522534; doi:10.1186/1756-3305-5-222)
Supplement: Additional file 2 — Figure S1. Temporal allele frequencies. Frequencies of alleles (above 0.05) across 15 loci are shown for the group of BU and OK samples (wet season of 2008 – dark blue, wet season of 2009 – blue, dry season of 2009 – light blue), and BV, BY, BZ and LI samples grouped together (dry season of 2010 – dark red, wet season of 2010 – light red). [file 1756-3305-5-222-S2.pdf]

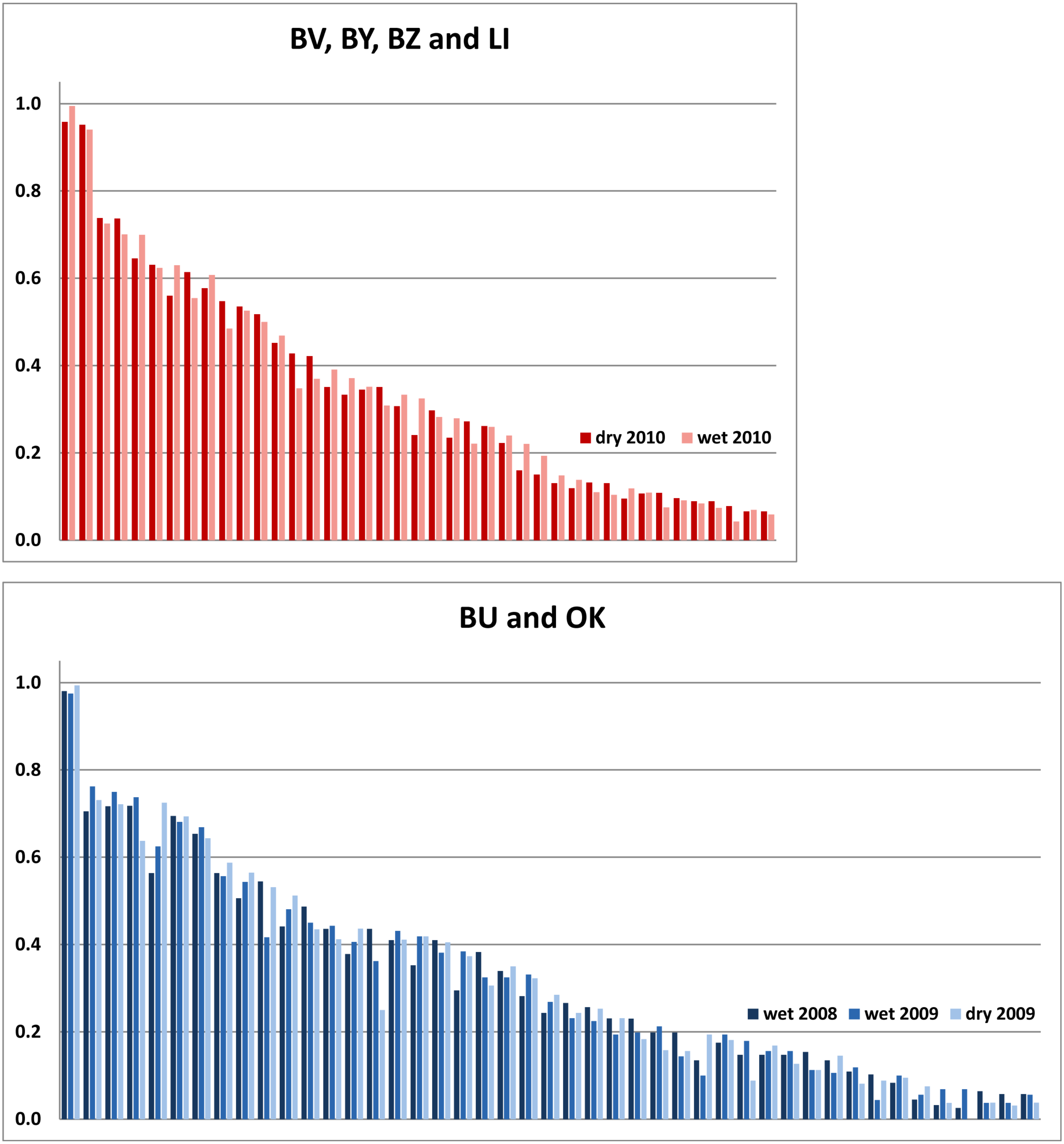

Figure S1. *Temporal allele frequencies.* Frequencies of alleles (above 0.05) across 15 loci are shown for the group of BU and OK samples (wet season of 2008 – dark blue, wet season of 2009 – blue, dry season of 2009 – light blue), and BV, BY, BZ and LI samples grouped together (dry season of 2010 – dark red, wet season of 2010 – light red).
