# Supplementary material for: The population structure of Glossina fuscipes fuscipes in the Lake Victoria basin in Uganda: implications for vector control
Source: Parasit Vectors. 2012 Oct 4;5:222. doi: 10.1186/1756-3305-5-222 (PMC3522534; doi:10.1186/1756-3305-5-222)
Supplement: Additional file 3 — Table S1. Fisher’s exact differentiation test . For each site or group of sites (BU-OK and BV-BY-BZ-LI), tsetse collected at different sampling times (wet and dry seasons; see Table 1) were tested for differences in allelic frequencies (genic differentiation) and genotypic frequencies. P (genic) and P (genotypic) – probability of non-differentiation. [file 1756-3305-5-222-S3.pdf]

Table S1. *Fisher's exact differentiation test*. For each site or group of sites (BU-OK and BV-BY-BZ-LI), tsetse collected at different sampling times (wet and dry seasons; see Table 1) were tested for differences in allelic frequencies (genic differentiation) and genotypic frequencies. P (genic) and P (genotypic) – probability of non-differentiation.

Population	P (genic)	P (genotypic)
**BU**	0.289	0.324
**OK**	0.561	0.641
**BU and OK**	0.082	0.135
**BV**	0.237	0.314
**BY**	0.198	0.263
**BZ**	0.630	0.782
**LI**	0.883	0.869
**BV, BY, BZ and LI**	0.292	0.376
