# Supplementary material for: The population structure of Glossina fuscipes fuscipes in the Lake Victoria basin in Uganda: implications for vector control
Source: Parasit Vectors. 2012 Oct 4;5:222. doi: 10.1186/1756-3305-5-222 (PMC3522534; doi:10.1186/1756-3305-5-222)
Supplement: Additional file 4 — Table S2. Pairwise FST values. FST values were computed for 4 clusters, averaged across 15 loci based on Weir and Cockerham [40]. FST values are reported in the lower diagonal and significance (α = 0.05) in the upper diagonal (‘-’: not significant; ‘+’: significant). [file 1756-3305-5-222-S4.pdf]

Table S2. *Pairwise F_{ST} values.* F_{ST} values were computed for 4 clusters, averaged across 15 loci based on Weir and Cockerham [40]. F_{ST} values are reported in the lower diagonal and significance ($\alpha = 0.05$) in the upper diagonal ('-': not significant; '+': significant)

	**Cluster 1**	**Cluster 2**	**Cluster 3**	**Cluster 4**
**Cluster 1**		+	+	+
**Cluster 2**	0.184		+	+
**Cluster 3**	0.191	0.124		+
**Cluster 4**	0.182	0.145	0.036	
