# Supplementary material for: The population structure of Glossina fuscipes fuscipes in the Lake Victoria basin in Uganda: implications for vector control
Source: Parasit Vectors. 2012 Oct 4;5:222. doi: 10.1186/1756-3305-5-222 (PMC3522534; doi:10.1186/1756-3305-5-222)
Supplement: Additional file 5 — Table S3. Pairwise FST values. FST values were computed for 14 sampling sites, averaged across 15 loci based on Weir and Cockerham [40]. FST values are reported in the lower diagonal and significance (α = 0.05) in the upper diagonal (‘-’: not significant; ‘+’: significant). Non-significant FST values are represented in bold italic type. [file 1756-3305-5-222-S5.pdf]

Table S3. *Pairwise F_{ST} values.* F_{ST} values were computed for 14 sampling sites, averaged across 15 loci based on Weir and Cockerham [40]. F_{ST} values are reported in the lower diagonal and significance ($\alpha = 0.05$) in the upper diagonal ('-': not significant; '+': significant). Non-significant F_{ST} values are represented in bold italic type.

	BU	OK	LI	BV	BY	BZ	BD	DB	KO	NS	EB	SS	KG	MA
**BU**		+	+	+	+	+	+	+	+	+	+	+	+	+
**OK**	0.023		+	+	+	+	+	+	+	+	+	+	+	+
**LI**	0.020	0.040		+	+	-	+	+	+	+	+	+	+	+
**BV**	0.028	0.057	0.009		+	-	+	+	+	+	+	+	+	+
**BY**	0.023	0.037	0.017	0.016		-	+	+	+	+	+	+	+	+
**BZ**	0.032	0.049	***0.004***	***0.010***	***-0.002***		+	+	+	+	+	+	+	+
**BD**	0.034	0.046	0.019	0.019	0.026	0.019		+	+	+	+	+	+	+
**DB**	0.113	0.127	0.089	0.081	0.107	0.105	0.087		+	+	+	+	+	+
**KO**	0.159	0.167	0.135	0.140	0.164	0.177	0.149	0.043		-	+	+	+	+
**NS**	0.171	0.167	0.140	0.137	0.162	0.176	0.140	0.035	***0.009***		+	+	+	+
**EB**	0.130	0.120	0.096	0.108	0.139	0.130	0.115	0.071	0.087	0.092		+	+	+
**SS**	0.167	0.177	0.158	0.158	0.197	0.222	0.185	0.202	0.182	0.190	0.136		+	-
**KG**	0.178	0.184	0.175	0.180	0.205	0.246	0.201	0.224	0.188	0.209	0.164	0.024		-
**MA**	0.174	0.184	0.173	0.181	0.209	0.248	0.204	0.236	0.208	0.223	0.169	***0.007***	***0.012***	
